# 

**Published:** 1859-11

**Authors:** 


					﻿CONTENTS.
When, and under what circumstances should teeth be extracted to secure a correct de-
velopment of permanent teeth. By James Taylor, M. D., D. D. S........ 201
The File................................................................. 215
An alarming case of Hemorrhage, from the Extraction of two Molar Teeth. By W. II.
Shadoan............................................................. 219
Thoughts on Fang Filling.	By Geo. Watt................................... 22
Correspondence........................................................... 22
A list of the Alumni of the Ohio College of Dental Surgery, from its organization to the
present time.......................................................... 230
Saint John’s Adjustable Dental Lathe.................................... 235
The Method of putting up the Vulcanite Base for Dentures................ 236
Directions for using Roberts’	Vulcanizing Apparatus..................... 237
Fang Filling.......................................................... 238
Selections.............................................................. 239
Submucous injection as a cure for the toothache of Pregnancy. By II. R. Storer, M. D. 239
Editorial,............................................................ 213
Popular Instruction..................................................... 243
A Practice to be Corrected............................................ 244
A “Crowners ’Quest”.................................................... 245
Four sets of Incisors.................................................. 247
Supernumerary Teeth............................................... .	248
A Case of Alveolar Abscess.............................................. 248
Scientific Artisan...................................................... 248
				

